# Flavone-Rich Fractions and Extracts from *Oroxylum indicum* and Their Antibacterial Activities against Clinically Isolated Zoonotic Bacteria and Free Radical Scavenging Effects

**DOI:** 10.3390/molecules26061773

**Published:** 2021-03-22

**Authors:** Patchima Sithisarn, Piyanuch Rojsanga, Pongtip Sithisarn

**Affiliations:** 1 Department of Veterinary Public Health, Faculty of Veterinary Medicine, Kasetsart University, Bangkok 10900, Thailand; fvetphs@ku.ac.th; 2 Department of Pharmaceutical Chemistry, Faculty of Pharmacy, Mahidol University, Bangkok 10400, Thailand; piyanuch.roj@mahidol.ac.th; 3 Department of Pharmacognosy, Faculty of Pharmacy, Mahidol University, Bangkok 10400, Thailand

**Keywords:** *Oroxylum indicum*, zoonotic infection, mechanism of action, flavone, HPLC, DPPH, scanning electron microscopy

## Abstract

*Oroxylum indicum* extracts from the seeds collected from Lampang and Pattani provinces in Thailand, and young fruits and flowers exhibited in vitro display antioxidant and antibacterial activities against clinically isolated zoonotic bacteria including *Staphylococcus intermedius*, *Streptococcus suis*, *Pseudomonas aeruginosa*, *β*-hemolytic *Escherichia coli* and *Staphylococcus aureus*. The orange crystals and yellow precipitates were obtained from the preparation processes of the seed extracts. The orange-red crystals from the seeds collected from Lampang province exhibited strong in vitro 2,2-diphenyl-1-picrylhydrazyl (DPPH) scavenging effects (EC_50_ value = 25.99 ± 3.30 μg/mL) and antibacterial effects on *S. intermedius* and *β*-hemolytic *E. coli* while the yellow precipitate from the same source exhibited only antioxidant activity. Quantitative analysis of phytochemicals in *O. indicum* samples by spectrophotometric and HPLC techniques showed that they contained different amounts of total phenolic, total flavonoid and three major flavones; baicalin, baicalein and chrysin contents. Young fruit extract, which contained low amounts of flavone contents, still promoted antibacterial effects against the tested bacteria with IC_50_ values lower than 1 mg/mL and MIC values between 4 to 10 mg/mL in *S. intermedius, S. aureus* and *S suis* while higher IC_50_ and MIC values against *P. aeruginosa* and *β*-hemolytic *E. coli* were found. From scanning electron microscopy, the extract of the young fruit of *O. indicum* promoted morphological changes in the bacterial cells by disrupting the bacterial cell walls, inducing leakage of the cellular content, and generating the abnormal accumulation of cells. The mechanism of action of the extract for this antibacterial effect may be the disruption of the cell membrane and abnormal cell aggregations. Regression analysis of the results suggests the correlation between total phenolic and total flavonoid contents and antioxidant and antibacterial effects. Baicalin was found to have a high correlation with an inhibitory effect against *β*-hemolytic *E. coli* while three unidentified peaks, which could be flavones, showed high correlations with an inhibitory effect against *S. intermedius*, *S. suis*, *P. aeruginosa* and *S. aureus*.

## 1. Introduction

Pheka (*Oroxylum indicum* (L.) Benth. ex Kurz) is a plant in the Bignoniaceae family which has been traditionally used for many medicinal purposes [1,2]. Young fruits of this plant can be consumed as vegetables while the mature seeds are the components in a traditional drink. Some flavonoids such as baicalein, biochanin A, oroxylin A, chrysin, apigenin, and their glycosides such as baicalein-7-*O*-diglucoside, scutellarein-7-*O*-glucopyranoside and chrysin-7-*O*-glucuronide have been recorded from various parts of this plant including the pods, the seeds, and the root bark [3,4,5,6,7,8,9,10]. The amount of flavonoids in the extracts from the seeds and the fruits of *O. indicum* were quantitatively analyzed using a high-performance liquid chromatography (HPLC) technique [11,12,13], while the chrysin contents in the seed samples collected in Thailand were quantitatively analyzed using a thin layer chromatographic (TLC) densitometry technique [14]. Alkaloids and triterpenes were also found in the seeds and pods [5,15]. The seed oil contained some fatty acids such as lauric, myristic, palmitic, stearic, oleic, and linoleic acids [1] while some phenolics such as anthraquinone, tannic acid and ellagic acid, alkaloids, and some phytosterols were reported in the leaves [3,4,16,17,18].

The leaf and fruit extracts of *O. indicum* were reported to reduce breast cancer cell growth, cell viability and cell migration [19]. Fresh pod 95% extract of this plant exhibited in vitro antioxidant activities and suppressed LPS plus IFN-c-activated reactive oxygen species generation in RAW264.7 macrophages [20]. The extract also showed potent anti-inflammatory action through suppressing nitric oxide and interleukin-6 secretion, due to its ability to scavenge intracellular reactive oxygen species and the alteration of signals relating to the activated lipid and protein determined by synchrotron radiation-based Fourier transform infrared (SR-FTIR) spectroscopy [20]. The hexane extract of the bark exhibited inhibitory effects on *Escherichia coli* and *Staphylococcus aureus* [21]. The 80% ethanol extract of *O. indicum* seeds exhibited an inhibitory effect on multidrug-resistant *S. aureus* [22]. Our previous reports demonstrated that ethanol extracts from *O. indicum* fruits and seeds exhibited in vitro antibacterial activities against clinically isolated *Staphylococcus intermedius* and *Streptococcus suis* and showed in vitro antioxidant activity as tested by the 2,2-diphenyl-1-picrylhydrazyl (DPPH) scavenging method [23]. Baicalin, baicalein and chrysin were found to be the active compounds [12,24]. Extracts from various plant parts of *O. indicum* including the leaves, flowers, seeds, stalks, and tissue-cultured plants and callus exhibited in vitro antioxidant activities with high total phenolic and total flavonoid contents and baicalin, baicalein, and chrysin as the major compounds [24]. The shoot extract from tissue-cultured plant at week 3 (cotyledon stage) contained high amounts of baicalin (14.78% *w*/*w* of the extract) while baicalein and chrysin were found in lower amounts [25]. Quantitative analysis of flavone contents by the HPLC method and investigation of in vitro antioxidant and antibacterial effects of some extracts and fractions from *O. indicum* samples and the study of mechanism of action using scanning electron microscopic technique were conducted.

## 2. Results

### 2.1. Determination of In Vitro Antioxidant Activity of O. indicum Samples Using DPPH Scavenging Method

Young fruit and flower extracts of *O. indicum* (OIYF and OIFL, respectively) appeared as dark brown semisolid extracts while *O. indicum* seed extracts from Lampang and Pattani provinces (OISL and OISP, respectively) appeared as semisolid dark green extracts. Yellow precipitates from *O. indicum* seed collected from Lampang and Pattani provinces (OIYL and OIYP, respectively) appeared as light yellow solid while OIRL and OIRP were orange-red crystals from *O. indicum* seeds collected from Lampang and Pattani provinces, respectively.

From the results (Table 1), the orange-red crystals OIRL and yellow precipitates OIYP exhibited the strongest 2,2-diphenyl-1-picrylhydrazyl (DPPH) scavenging activities with EC_50_ values of 25.99 ± 3.30 and 29.16 ± 0.07 μg/mL, respectively. Seed extract OISP, yellow precipitates OIYL and orange-red crystals OIRP also promoted good DPPH scavenging effects while seed extract OISL, young fruit extract OIYF and flower extract OIFL promoted lower DPPH scavenging effects. OIRL, OIYP and OIYL which promoted strong DPPH scavenging effects, contained high total phenolic and total flavonoid contents (>6 g% in the extracts).

### 2.2. Determination of Total Phenolic Content in O. indicum Samples Using the Folin-Ciocalteu Method and Determination of Total Flavonoid Content in O. indicum Samples Using the Aluminium Chloride Method

As shown in Table 1, most *O. indicum* samples contained high amounts of total phenolic and total flavonoid contents, except the flower extracts which contained a low amount of total flavonoid content (<1 g% QE).

### 2.3. Analysis of Flavone Contents in O. indicum Samples by HPLC

**Figure 1:**
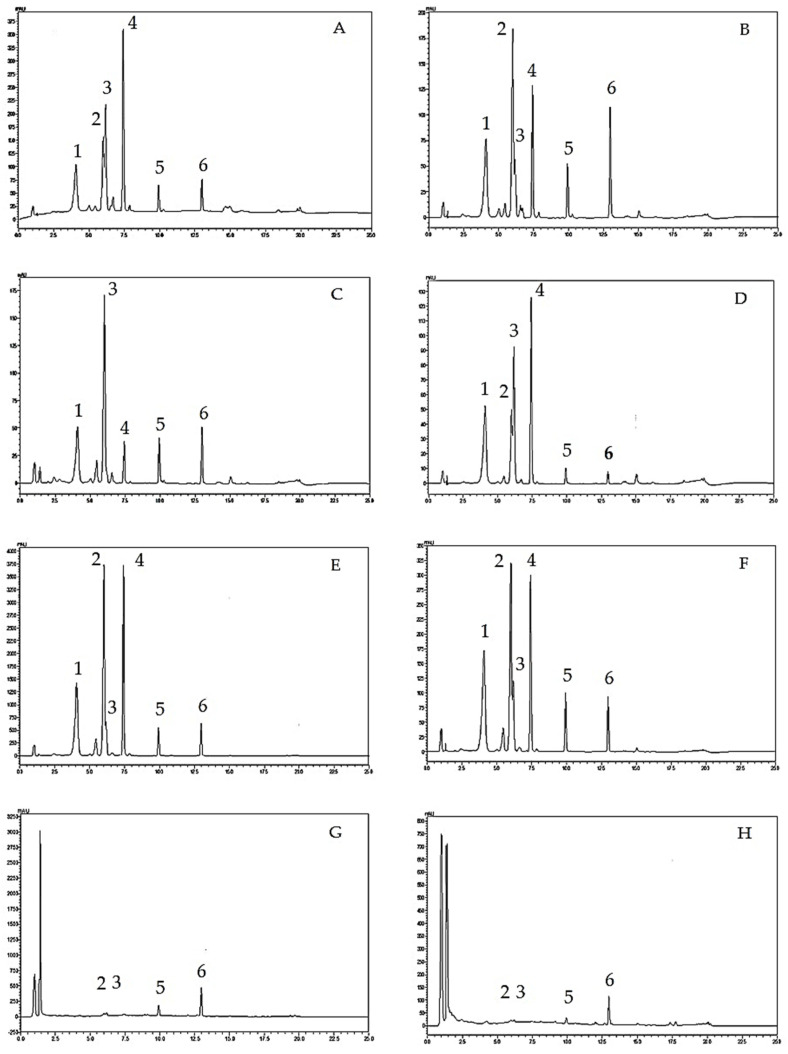
HPLC chromatograms of *Oroxylum indicum* samples; (**A**) = OIRL(orange-red crystals from *O. indicum* seed collected from Lampang province), (**B**) = OIYL (yellow precipitates from *O. indicum* seed collected from Lampang province), (**C**) = OISL (*O. indicum* seed extracts from Lampang province), (**D**) (OIRP = orange-red crystals from *O. indicum* seed collected from Pattani province), (**E**) = OIYP (yellow precipitates from *O. indicum* seed collected from Pattani province), (**F**) = OISP (*O. indicum* seed extracts from Pattani province), (**G**) = OIYF (*O. indicum* young fruit extract), and (**H**) = OIFL (*O. indicum* flower extract). HPLC peaks; 1 = compound Rt 4, 2 = compound B0, 3 = baicalin, 4 = compound Rt 7, 5 = baicalein, 6 = chrysin.

Regarding the system suitability parameters, tailing factors of all three standard compounds were less than 2, theoretical plate numbers were in the range of 10,000 to 90,000% relative standard deviation (RSD) values of the peak area were less than 2 and the standard deviation (SD) values of the retention times of each peak were less than 1, suggesting the suitability of the analytical method. Figure 1 shows the HPLC chromatograms of *O. indicum* samples.

**Figure 2:**
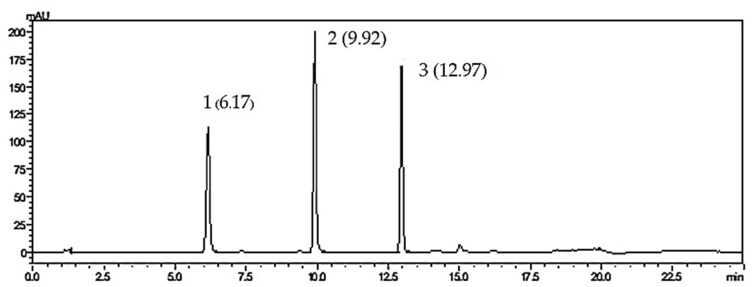
HPLC chromatograms of standard compounds; 1 = baicalin, 2 = baicalein, 3 = chrysin.

As shown in Figure 1, HPLC analysis of *O. indicum* samples showed specific chromatographic fingerprints with the presences of the peaks that corresponded to baicalin, baicalein and chrysin at retention times of 6.17, 9.92 and 12.97 min, respectively, according to standard mixture HPLC chromatogram in Figure 2. However, in most *O. indicum* samples, there was a peak (peak B0) that appeared at the retention time of 6.00 min before the peak of baicalin, except in the seed extract of *O. indicum* collected from Lampang province (OISL) which did not show the presence of this peak (B0). Moreover, there were other major peaks at retention times of 4.09 and 7.42 min (peak Rt 4 and peak Rt 7, respectively) in most *O. indicum* samples except in *O. indicum* flower extract (OIFL) which did not have both peaks and *O. indicum* young fruit extract (OIYF), which did not have peak Rt 4. Table 2 summarizes the HPLC retention times, UV spectra, previous reported MS data and chemical structures of six major peaks in *O. indicum* extracts and fractions.

Quantitative analysis of the three flavones; baicalin, baicalein and chrysin, by HPLC in the *O. indicum* samples showed that OISL and OIRL contained the highest amounts of baicalin (>6 g% in the extracts). Other *O. indicum* samples from the seeds contained moderate amounts of baicalin while the extracts from the young fruits and the flowers of *O. indicum* contained low amounts of this compound (<1 g% in the extracts). Baicalein and chrysin were found in lower amounts than baicalin in all *O. indicum* samples (not more than 3 g% in the extracts). The total amounts of baicalin and compound B0 (calculated as baicalin) are also shown in Table 1. OISP, OIYP and OIRL showed high cumulative amounts of baicalin and compound B0 (>10 g% in the extracts) which correlate to their high antioxidant activities. The results suggest that compound B0 should be responsible for the antioxidant activities of the *O. indicum* samples.

### 2.4. Determination of In Vitro Antibacterial Activity of O. indicum Samples Using Disc Diffusion Assay

*Oroxylum indicum* seed samples collected from Lampang province including orange-red crystals, yellow precipitate and extract (OIRL, OIYL and OISL, respectively) and young fruit extract (OIYF) were determined for antibacterial effects against clinical isolated zoonotic bacteria including *S. intermedius*, *S. suis*, *P. Aeruginosa,* and *β*-hemolytic *E. coli* and *S. aureus* using the disc diffusion method. As shown in Table 3, both seed extracts (OISL) and young fruit extracts (OIYF) contained low amounts of flavone contents but still promoted antibacterial effects against all of the tested bacteria. Orange-red crystals of *O. indicum* from Lampang province (OIRL) showed strong inhibitory effects on *β*-hemolytic *E. coli* and a weak inhibitory effect against *S. intermedius* while the yellow precipitate (OIYL) showed no inhibitory effect against all of the tested bacteria. Since OIYF showed a reliable antibacterial activity to every pathogen, and the young fruit of *O. indicum* is a part of the plant considered appropriate in terms of accessibility and practicality for quality control, it was the first priority among extracts submitting for determination of half-maximal inhibitory concentrations (IC_50_) and minimum inhibitory concentrations (MICs).

### 2.5. Determination of the Half-Maximal Inhibitory Concentrations (IC_50_) and the Minimum Inhibitory Concentrations (MICs)

The half-maximal inhibitory concentrations (IC_50_) and the minimum inhibitory concentrations (MICs) of OIYF to each pathogenic bacterium were determined. The young fruit extract of *O. indicum* (OIYF) showed effectiveness in inhibiting the five clinically important bacteria. In gram positive bacteria, *S. aureus*, *S. intermedius*, and *S. suis,* they were subdued by OIYF more profoundly compared to clinically important gram negative bacteria, *β*-hemolytic *E. coli*, and *P. aeruginosa*. OIYF showed the lowest IC_50_ and MIC values against *S. aureus* at 0.40 ± 0.00 mg/mL and 4.67 ± 0.04 mg/mL, respectively. Corresponding results were found in *S. intermedius* and *S. suis* at IC_50_ and MIC values 0.81 ± 0.04 mg/mL and 5.17 ± 1.23 mg/mL in *S. intermedius*, 0.96 ± 0.46 mg/mL and 10.54 ± 1.00 mg/mL in *S. suis* accordingly. The results suggested similar mechanisms of antibacterial activity of OIYF toward these three zoonotic bacteria, or at least one stated association of the chemical–pathogen interactions involved in particular characteristics of gram positive bacterial cell wall and cell membrane dysfunction. This may be attributed to either direct damage to the cytoplasmic membrane or indirect damage effected through autolysis/weakening of the cell wall and consequent osmotic lysis. Interestingly, inhibitory activity of the extract to *β*-hemolytic *E. coli* and *P. aeruginosa* were shown to be 16.35- and 8.93-fold of IC_50_ values and 4- and 9.8-fold of MIC values compared to *S. Aureus,* suggesting various mechanisms of action in the antibacterial activities of the extract [27]. Table 4 showed minimum inhibitory concentrations (MICs) and half-maximal inhibitory concentration (IC_50_) of *O. indicum* young fruit extract (OIYF) against clinical isolated zoonotic bacteria. Chemico–pathogen interactions and the significance of the *O. indicum* flavone-contained extracts attributed to bacterial cytoplasmic membrane integrity and membrane alteration were then determined using scanning electron microscopy.

### 2.6. Evaluation of Antibacterial Mechanism of Action of O. indicum by Scanning Electron Microscopic Technique

**Figure 3:**
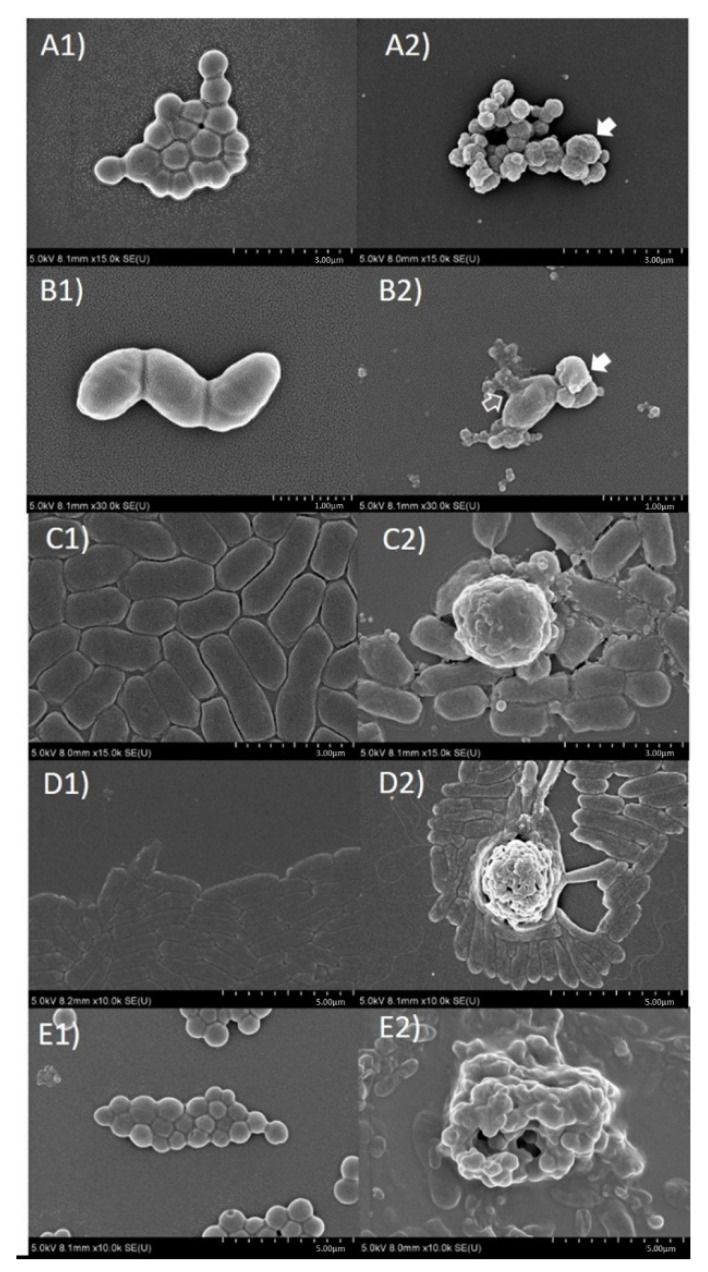
Scanning electron microscopic micrograph (SEM) of clinically isolated bacteria after treatment with young fruit extract of *O. indicum* (OIFY) at the concentration of 5 mg/mL; (**A1**) = *Staphylococcus intermedius* without OIYF treatment, (**A2**) = *Staphylococcus intermedius* with OIFY treatment; (**B1**) = *Streptococcus suis* without OIYF treatment, (**B2**) = *Streptococcus suis* with OIYF treatment, (**C1**) = *β*-hemolytic *Escherichia coli* without OIYF treatment, (**C2**) = *β*-hemolytic *Escherichia coli* with OIYF treatment, (**D1**) = *Pseudomonas aeruginosa* without OIYF treatment, (**D2**) = *Pseudomonas aeruginosa* with OIYF treatment, (**E1**) = *Staphylococcus aureus* without OIYF treatment, (**E2**) = *Staphylococcus aureus* with OIYF treatment. (**A2**,**B2**) White arrows = cell fusion, (**B2**) Grey arrow = cellular content leakage.

After the young fruit extract of *O. indicum* (OIFY) at a concentration of 5 mg/μL was incubated with 3 × 10^6^ CFU/well in MHB of each clinical isolated bacteria; *S. intermedius*, *S. suis*, *β*-hemolytic *E. coli*, *P. aeruginosa* and *S. aureus* at 37 °C for 15 h, a significant morphological damage and agglutination of the cells treated with OIFY was clearly shown (Figure 3A2–E2) compared to the untreated cells (Figure 3A1–E1). The pathogens had decreased in size and changed in shape, seen in cell wrinkle and shrinkage. They also created signs of cellular membrane disruption, leakage of cellular contents, cell fusion and agglutination. The results suggest that the mechanisms of action of *O. indicum* young fruit extract on these clinically important pathogens could be on the bacterial cell membrane, extracellular proteins or other regulatory protein-induced cell membrane disruption and cell lysis.

### 2.7. Evaluation of Correlations between Antioxidant and Antibacterial Activities and Phytochemical Contents

Table 5 shows the correlation between the antioxidant and antibacterial activities and the total phenolic contents, total flavonoid contents and HPLC peak areas of flavones in *O*. *indicum* samples including baicalin, baicalein, chrysin and also the HPLC peak areas of three unidentified compounds; compound at retention time = 6.00 min (compound B0), compound at retention time = 4.09 min (compound Rt 4) and compound at retention time 7.42 min (compound Rt 7). Total phenolic and total flavonoid contents showed high correlations with the inhibitory effects against *S*. *suis*, *P*. *aeruginosa* and *S*. *aureus* (R values > 0.8) while only the total flavonoid content showed a high correlation with the inhibitory effect against *S*. *intermedius* (R value = 0.925). Both total phenolic and total flavonoid contents showed a low correlation with the inhibitory effects against *β*-hemolytic *E*. *coli*. Peak areas of all three unidentified compounds, compound B0, compound Rt 4 and compound Rt 7 and total peak area of compound B0 and baicalin showed high correlations with inhibitory effects against *S*. *suis*, *P*. *aeruginosa* and *S*. *aureus* while only the peak area of compound B0 showed a high correlation with inhibitory effects against *S. intermedius*. The only peak area of baicalin showed a high correlation with inhibitory effects against *β*-hemolytic *E*. *coli* while other peak areas of other compounds in *O*. *indicum* samples showed low or moderate correlations. The results suggest that most tested bacteria except *β*-hemolytic *E*. *coli* are susceptible to most *O*. *indicum* samples, due to the effects of their chemical constituents. Peak areas of baicalein and chrysin exhibited a low to moderate correlation with inhibitory effects against all of the selected clinical isolated bacteria. Regarding the antioxidant activities, total phenolic and total flavonoid contents and the peak area of baicalin exhibited high correlations with the DPPH scavenging effects, peak areas of baicalein, compound B0, compound Rt 4 and compound Rt 7 also exhibited moderate correlations while the chrysin content exhibited a low correlation.

## 3. Discussion

*O. indicum* seed, young fruit and flower extracts, orange-red crystals and yellow precipitate promoted in vitro DPPH scavenging effects and antibacterial activities on clinical isolated bacteria including *S. intermedius*, *S. suis*, *P. aeruginosa*, *β*-hemolytic *E. coli* and *S. aureus*. Most *O. indicum* samples contained high amounts of total phenolic and total flavonoid contents together with the presence of three flavones, baicalin, baicalein and chrysin and another three unidentified compounds, compound B0, compound Rt 4 and compound Rt 7. The generation of orange-red crystals and yellow precipitate could be found from both *O. indicum* seeds collected in Lampang province in 2019 and from Pattani province in 2016. Orange-red crystals from *O. indicum* seeds collected from both provinces contained very similar amounts of baicalin content as the seed extracts from the same sources while yellow precipitates contained lower amounts of baicalin. Orange-red crystals and the seed extract from *O. indicum* seeds collected in Lampang province (OIRL and OISL, respectively) contained higher baicalin contents than the orange-red crystal and the seed extract from *O. indicum* seeds collected from Pattani province (OIRP and OISP, respectively). These results suggest that the collection locations and the storage time of the plant material could affect the phytochemical contents of *O. indicum* seeds. Young fruit extract (OIYF), which contained low amounts of flavone contents, still promoted antibacterial effects against tested bacteria suggesting that there could be other phytochemicals responsible for these antibacterial effects. The possible process of generation of these orange-red crystals and yellow precipitates could be the saturation of flavones in the extraction solution during the evaporation process resulting in the precipitation of enriched flavone mixtures in different forms. However, factors related to their generation or to the specific ratios of their chemical composition are still unclear.

Total phenolic and total flavonoid contents and peak areas of baicalin exhibited a significant correlation to antioxidant activity; peak areas of baicalein, compound B0, compound Rt 4 and compound Rt 7 showed moderate correlations while chrysin content showed a low correlation. The results correspond to our previous studies, which revealed that total phenolic and total flavonoid contents could be responsible for the antioxidant activities of *O. indicum* extracts while baicalin and baicalein were found to be the active compounds [24]. Total phenolic, total flavonoid and peak areas of compound B0, compound Rt 4 and compound Rt 7 showed high correlations with the inhibitory effects against *S. suis*, *P. aeruginosa* and *S. aureus*. Total flavonoid content and peak area of compound B0 exhibited a high correlation with the inhibitory effects against *S. intermedius*. Only the peak area of baicalin exhibited a high correlation with the inhibitory effect against *β*-hemolytic *E. coli*.

In this study, the seed extract from *O. indicum* collected from Lampang province and the young fruit extract promoted in vitro antibacterial effects on all tested bacteria. The results correspond to our previous study which reported that the fruit ethanol extract of *O. indicum* promoted high inhibitory effects on these bacteria [12]. Orange-red crystal from Lampang province was found to exhibit high inhibitory effects on *β*-hemolytic *E. coli,* which could be related to the high amount of baicalin content that was previously reported to promote a significant inhibitory effect on this bacterium [12]. Our previous report suggested that there could be a specific ratio or range of active flavonoids in *O. indicum* extracts necessary to promote a strong antibacterial effect or there could be other active constituents that promote antibacterial activity in *O*. *indicum* extracts [12]. These active compounds could be compound B0, compound Rt 4 and compound Rt 7 as their peak area was highly correlated to the antibacterial effect in this study. The previous report suggested that compound B0 could be orexin A while compound Rt 4 could be orexin B and compound Rt 7 could be chrysin-7-*O*-glucuronide [13]. Orexin A was reported to promote antioxidant, anticancer, antibacterial and antidiabetic effects [28,29], while oroxin B showed anti-lymphoma effect [30], inhibitory effects to the hemolytic activity [31] of α-hemolysin and anticancer effects [32,33]. Chrysin-7-*O*-glucuronide also exhibited antioxidant activity [34]. Our study suggests that these compounds could be responsible for the antioxidant and antibacterial activities of the *O*. *indicum* samples.

However, the conditions of orange-red crystal and yellow precipitate generations are still unclear. There could be various possible phenomena including the co-crystallization of the compositional flavones in the evaporating extract solution in different ratios or the forming rates resulting in the precipitation of a yellow precipitate or the generation of orange-red crystals. Co-crystals have been reported as multiple component crystals in which all components are solid under ambient conditions whilst in their pure form [35]. One report suggested the co-crystallization of naringenin with 27 co-formers, which led to the formation of a salt and four co-crystals [36]. Several new forms of quercetin in the form of solvates and co-crystals were synthesized [37]. Solvated quercetin was generated by quercetin crystallized with two water molecules that participate in an extended hydrogen bonding network while the quercetin dimer was formed by the hydroxyl (OH) and the adjacent carbonyl moiety hydrogen bonding [37]. In this report, two different co-crystal quercetins; co-crystals I and II with caffeine and iso-nicotinamide could be formed during the slow evaporation [37]. Nicotinamide and picolinic acid co-crystals of quercetin were also reported [38]. The properties of the solid binary system between quercetin and nicotinamide including the melting point, solubility and physical morphology were reported to be different from the physical mixture of quercetin-nicotinamide [39]. These studies suggest that co-crystallization could be occurring between flavonoids and the different molecules that have functional groups which could be forming bonds with them, then altering the properties of the compounds. Therefore, in this study, three major flavones, baicalin, baicalein and chrysin, could be forming co-crystals between themselves or with other molecules in the extract solution during the evaporation process. Another possible phenomenon is crystallization in different hydrated forms. Wogonin, a flavone compound, can be formed as two distinct hydrated crystal forms, yellow block-shaped crystals of wogonin monohydrate and yellow needle-shaped crystals of wogonin sesquihydrate [40]. This report suggested that the formation of wogonin crystals could be affected by the uncertainty in the water content in the atmosphere and also by contamination with the phase of alternate hydrate stoichiometry [40]. These forms of wogonin were found to affect the solubility and bioavailability of this compound in an animal model [41]. The final phenomenon that could affect the precipitation of yellow precipitate and orange-red crystal of *O*. *indicum* seed extracts is the formation of different polymorphisms of flavonoids. Two crystalline components obtained from the gel filtration chromatographic separation of hexane extract from *Melicope ellyrana* fruits were found to be different polymorphs of the same flavonoid [42]. These two polymorphs showed significant conformational differences, particularly in the enyloxy side chain, while only one (polymorph A) shows intermolecular hydrogen bonding [42]. Previous reports suggest the possible factors related to the formation of orange-red crystals and yellow precipitates from *O. indicum* seed extracts, which affects their biological activities and physical properties. In this study, the ratios between baicalin and compound B0 are various in orange-red crystals, yellow precipitates and seed extracts of *O*. *indicum* from both Lampang and Pattani provinces. In orange-red crystals, baicalin contents are moderate to high while the differences between the amounts of baicalin and compound B0 are not high. In yellow precipitate, baicalin contents are always low while the amounts of compound B0 are high. In seed extracts, the amount of baicalin is moderate to high while compound B0 could be found in a high amount or could not be found. Comparison between these extracts and enriched fractions suggests that both baicalin and compound B0 play an important role in DPPH scavenging effects, while baicalin is mainly responsible for antibacterial effects, and a significant ratio between baicalin and B0 in the compounds has been observed. Further study should be conducted.

As for the mechanism of antibacterial activity, it was found that *O. indicum* young fruit extract caused the changes in bacterial cellular morphology and increase in cell lysis of the tested pathogens, *S. intermedius*, *S. suis, β*-hemolytic *E. coli*, *P. aeruginosa* and *S. Aureus,* suggesting the action on the integrity and function of the cell membrane or extracellular proteins, resulting in the disruption of bacterial cell wall/membrane and in destruction of the bacterial cells. The extract also caused the abnormal aggregations in some bacteria including *S. intermedius*, *S. suis* and *S. aureus*. This is the first display of the mechanism of action of *O. indicum* young fruit extract against these important pathogens that could act via cell membrane structure loss of integrity and cell membrane function alteration. Some phytochemicals including phenolics and flavonoids, mainly flavones, alkaloids, terpenes and fatty acids, have been found in various parts of *O. indicum* [3,4,5,6,7,8,9,10,15,16,17,18]. These compounds could act together by different mechanisms of action to promote antibacterial effects. Two major mechanisms of interaction of flavonoids with the lipid bilayer of microorganisms were previously reported; the partition of the non-polar compounds in the hydrophobic interior of the membrane, and the formation of hydrogen bonds between the hydrophilic flavonoids and the polar head groups of lipids at the membrane interface [43]. Moreover, nonspecific interactions of flavonoids with phospholipids can induce structural changes of the membrane such as thickness and fluctuations which indirectly modulate the function of membrane proteins, as well as influence the pharmacological properties of flavonoids themselves [44]. Some flavones including acacetin and apigenin were reported to cause destabilization of the membrane structure by disordering and disorientation of the membrane lipids and induced leakage from the vesicle [45]. There are studies suggesting that the differences in the number and distribution of hydroxyl groups, the polymerization degree, and the presence of a methoxy groups in the C ring affect the type of interactions between different flavonoids and lipid bilayers [46]. There are controversial suggestions about the polarity of flavonoids and their effects on membrane disruption, including a suggestion that flavonoids lacking hydroxyl groups on their B rings are more active against microbial membranes than those with the hydroxyl groups [47], while other studies suggested that lipophilic flavonoids which are highly hydroxylated can be more disruptive to membrane structure [27,48,49,50]. Another report states that flavonoids cause bacterial aggregation by their partial lysis, which leads to membrane fusion, and consequently reduces the active nutrient uptake via a smaller membrane area [27]. Another suggests that flavones such as 6-amino-flavone, 6-hydroxy-flavone, apigenin and chrysin had inhibitory effects on *E. coli* O157:H7 biofilm formation [51]. Some flavones including 5,6,7,4′,5′-penta-hydroxy-flavone, and 5-hydroxy-4′,7-dimethoxyflavone were reported to downregulate the malonyl CoA-acyl carrier protein trans-acylase fabD (MCATs) that regulates bacterial-type II fatty acid synthase (FAS-II) which related to inhibition of cell envelope synthesis [52]. Baicalein induced the damage of peptidoglycan, an essential component of the bacterial cell wall [53], while chrysin showed inhibitory effect on DNA gyrase from *E. coli* [54]. A molecular docking study suggested that flavonoids inhibit the DNA supercoiling by competitively interacting with the ATP binding site of the DNA gyrase B subunit (GyrB) [55,56]. Flavones and flavonols were previously suggested as pharmacophores with nucleic acid binding capacity, especially by helicase inhibition [27]. A flavone luteolin inhibited the replicative helicases such as the DnaB and RecBCD helicase/nuclease of *E. coli* [57]. Baicalein was also reported as one of the most effective inhibitors of *E. coli* F1FO ATPase [58]. Moreover, flavonoids have been proposed as resistance modifying agents (RMAs) which could promote synergy and additive effects with antibiotics by inhibition of efflux pumps or antibiotic degrading enzymes and membrane permeabilization [59]. Baicalein has been reported to promote synergistic effects with tetracycline against methicillin-resistant *Staphylococcus aureus* (MRSA) [53], and with penicillin and amoxicillin against MRSA and penicillinase-producing *S. aureus* (PPSA), respectively [60] and with cloxacillin against *S. aureus* DMST 20,651 [61]. Baicalein at the concentration of 64 μg/mL acted as efflux pump inhibitors against *Mycobacterium smegmatis* and *Candida albicans* [62,63]. At the concentration of 16 μg/mL, it could also reverse the ciprofloxacin resistance of MRSA by inhibition of NorA efflux pump [64]. Previous studies suggest that baicalein and chrysin, two of the major flavones in *O. indicum* along with other phenolics and flavonoids, could promote different mechanisms of action to inhibit bacteria growth. From our study, baicalin and compound B0 which has been proposed as oroxin A showed high correlation in antibacterial effects with most tested bacteria. The major mechanism of action should be the formation of hydrogen bonds between the hydrophilic parts of flavonoids and the polar head groups of lipids at the membrane interface so that bacterial membrane depolarization and membrane potential alteration can take place [65], though the novel mechanism of bacterial virulence protein inhibition compromising bacterial transmembrane heptameric channels, causing cell death and the lipophilicity of the flavone components, have been discussed [31,66]. The significance of the ratio between baicalin and compound B0 contents should be noted. OIYL, which showed significant difference between baicalin and compound B0 contents, promoted no antibacterial effect while OIYF, which contained lower amounts of both baicalin and compound B0 contents, exhibited decent antibacterial effects. The antibacterial effects of OIYF could be due to the suitable ratio between baicalin and compound B0 contents. Total flavonoid and total phenolic contents also showed high correlation with antibacterial effects, which could be due to the multi-targeting effects of various flavonoids and phenolics in *O. indicum*. For DPPH scavenging effects, it was clear that the antioxidant effects come from the proton donating abilities of the phytochemicals in *O. Indicum*. Therefore, the amounts of total phenolic, total flavonoid and compounds with proton donating functional groups, such as hydroxyl groups, play an important role in the antioxidant effects. Further study of the mechanism of action of *O. indicum* should be conducted in the future.

## 4. Materials and Methods

### 4.1. Chemicals

Deionized water was obtained by using a water purification system from Thermo Scientific Co. (Waltham, MA, USA). Standard baicalin and chrysin, at a pharmaceutical grade, were purchased from TRC (North York, ON, Canada). Acetonitrile, ascorbic acid, baicalein, DPPH and quercetin at an analytical reference grade, were purchased from Sigma-Aldrich (St. Louis, MO, USA). Aluminum chloride, Folin-Ciocalteu reagent and phosphoric acid were purchased from Merck (Darmstadt, Germany). Gallic acid at an analytical reference grade, was purchased from TOKYO chemical industry Co., Ltd. (Tokyo, Japan). Sodium carbonate was purchased from Ajax Finechem Pty. Ltd. (Sydney, Australia). 

### 4.2. Plant Materials

The mature seeds of *O. indicum* were collected from Lampang province in 2019 and from Pattani province in 2016. The fresh young fruits of *O. indicum* were collected from Nakhon Pathom and Chiang Rai provinces in 2019 while the flowers were collected from Chiang Rai province in 2019. Plant samples were identified by Associate Prof. Pongtip Sithisarn. Plant samples were cleaned and dried in a hot air oven (Memmert, Schwabach, Germany) at 60 °C, then ground using an electric milling machine (Ika-Werke, Staufen, Germany) (20 mesh sieve).

### 4.3. Plant Extract Preparations

Each sample of *O. indicum* from different plant parts was separately extracted by maceration using 95% ethanol (plant:solvent ratio 1:20 *w*/*v*). Each powdered plant sample was separately macerated with 95% ethanol using an electric flask shaker (Wisd Laboratory Instruments, Wertheim, Germany) for 6 h. The extraction solution was filtered after it was stored for 12 h. Each extraction process was repeated three times. The extraction solutions were then combined, filtered, and evaporated using a water bath to yield the dried extracts. Both young fruit and flower extracts (OIYF and OIFL, respectively) were obtained. The solutions of seed extract of *O. indicum* from Lampang and Pattani provinces were then brought by the separation process to the yellow precipitate and the orange-red crystals.

### 4.4. Separation of Yellow Precipitate and Orange-Red Crystal from the Seed Extracts of O. indicum 

During the evaporation of both extract solutions of *O. indicum* seeds, collected from Lampang and Pattani provinces using a rotary evaporator, there was precipitation of a yellow precipitate. This precipitate was separated and collected (OIYL and OIYP for the yellow precipitate from *O. indicum* seeds collected from Lampang and Pattani provinces, respectively). Some parts of this yellow precipitate were then turned into orange-red crystals after they were dried, so they could be separated into OIRL and OIRP for the orange-red crystals from *O. indicum* seeds collected from Lampang and Pattani provinces, respectively. The rest of the extract solutions from each type of *O. indicum* seeds were then dried and a semisolid dark green extract was obtained from the *O. indicum* seeds from Lampang and Pattani provinces (OISL and OISP, respectively).

### 4.5. Determination of In Vitro Antioxidant Activity of O. indicum Samples Using DPPH Scavenging Method

The 2,2-diphenyl-1-picrylhydrazyl (DPPH) was dissolved in methanol to prepare the DPPH solution at a concentration of 207 μM. The DPPH solution (100 μL) was added to each *O. indicum* sample solution at various concentrations ranging from 5–640 μg/mL at the same volume (100 μL). The mixture was mixed and kept in the dark for 15 min. The absorbance of each reaction solution was determined at a wavelength of 515 nm using a micro-plate reader. The percentage of inhibition for each reaction was then calculated, and EC_50_ values (μg/mL) were calculated from the linear equation from the curve between the percentage of inhibition and the solution’s concentration. Each experiment was conducted in triplicate. The EC_50_ value of each sample was expressed as the mean ± SD. The assays were performed as previously described [24].

### 4.6. Determination of Total Phenolic Content in O. indicum Samples Using the Folin-Ciocalteu Method

The Folin-Ciocalteu assay was carried out according to the method previously described [24] with gallic acid as the standard solution. A Folin-Ciocalteu reagent was added to each *O. indicum* sample or standard gallic acid solution, then the solution was kept in the dark at room temperature for 90 min. After that, a 20% *w/v* sodium carbonate solution was added to each mixture. The absorbance of the reaction solution was determined at a wavelength of 765 nm using a micro-plate reader. The total phenolic compound was calculated from the standard curve of gallic acid and was expressed as g gallic acid equivalent per 100 g extract (g% GAE). Each experiment was carried out in triplicate. The average total phenolic content and standard deviation were then calculated.

### 4.7. Determination of Total Flavonoid Content in O. indicum Samples Using the Aluminium Chloride Method

An aluminum chloride determination method was employed using quercetin as the standard solution [24]. An aluminum chloride solution (10% *w*/*v*) was added to each *O. indicum* sample or standard quercetin solution. The mixture was kept at room temperature for 30 min. The absorbance of the reaction solution was determined at a wavelength of 415 nm using a micro-plate reader. The total flavonoid content was calculated from the standard curve of quercetin and was expressed as g quercetin equivalent per 100 g extract (g% QE). Each experiment was carried out in triplicate. The average total flavonoid content and standard deviation were then calculated.

### 4.8. Analysis of Flavone Contents in O. indicum Samples by HPLC

The contents of three flavones in the *O. indicum* samples were quantitatively analyzed using the validated HPLC method modified from the previous report [12]. A HPLC analysis was performed using a Shimadzu LC-10ADVP system (Shimadzu, Kyoto, Japan) equipped with a diode array detector (DAD) SPD-M10AVP and a column heater (Shimadzu, Kyoto, Japan). An X-terra C18 column (150 mm × 3.9 mm, 5 μm particle size) from Waters (Milford, MA, USA) was used. Gradient elution was performed with a water −0.01% phosphoric acid (solvent A) and acetonitrile (solvent B) at a constant flow rate of 1.2 mL/min. Column temperature was 40 °C with an injection volume of 20 μL; detection was performed at 285 nm. The system suitability of the analytical method, including the tailing factor, theoretical plate, %RSD of the peak area and the SD of the retention time, was also investigated.

### 4.9. Clinical Isolated Bacterial, Bacterial Culture, and Culture Media 

Clinical isolates of *S. intermedius*, *S. suis*, *P. aeruginosa*, *β*-hemolytic *E. coli* and *S. aureus* were obtained from the Microbiological Laboratory, Veterinary Diagnostic Center, Faculty of Veterinary Medicine, Kasetsart University, Nakhon Pathom, Thailand. The bacteria strains were isolated and characterized using differential bacterial culture and biochemical assays for clinical samples according to the standard method of Baron et al. [66]. *S. Suis*, *P. aeruginosa*, *β*-hemolytic *E. coli*, and *S. aureus* were maintained in Microbank Cryovials and kept at −80 °C. *S. intermedius* was maintained in skimmed milk at −40 °C until use. Prior to any particular testing, each bacterial strain was maintained on a blood agar plate and incubated for 18–24 h at 37 °C. Blood agar was obtained from Veterinary Diagnostic Center, Faculty of Veterinary Medicine, Kasetsart University. Mueller-Hinton agar and Mueller-Hinton broth were purchased from Oxoid, Thermofisher Inc. (St.Louis, MO, USA).

### 4.10. Determination of In Vitro Antibacterial Activity of O. indicum Samples Using Disc Diffusion Susceptibility Test 

Disc diffusion method was used for antibacterial activity of *O. indicum* extracts from different parts of the plants and the location collected as described earlier: orange-red crystal, yellow precipitates from fruits and the young fruit extract, according to Kirby-Bauer disc diffusion method and CLSI [67,68]. Five clinically important pathogenic bacteria were examined; *S. intermedius*, *S. suis*, *P. aeruginosa*, *β*-hemolytic *E. coli,* and *S. aureus*. A stock solution of each *O. indicum* extract in DMSO was prepared to produce a final concentration of 200 mg/mL. The stock solution was then diluted to concentrations of 3.125, 6.25, 12.5, 25, 50 and 100 mg/mL. Twenty microliters of each dilution was impregnated into sterile, blank discs 6-mm in diameter. After the extract was spotted, the discs were allowed to dry to ensure precise impregnation. Twenty microliters of DMSO was used as negative control. All fully dried discs were applied on the corresponding bacterial lawn. Mueller-Hinton agar (*S. intermedius, P. aeruginosa, β*-hemolytic *E. coli, and S. aureus*) or blood agar (*S. Suis*) was used. The density of the bacterial culture used in lawning was adjusted to 0.5 McFarland standard in Mueller-Hinton broth using a turbidimeter (Biosan, Riga, Latvia). The positive controls were standard antibiotic discs, amoxicillin/clavulanic acid 30 μg, doxycycline 30 μg, and sulfamethoxazole-trimethoprim 25 μg (Sigma-Aldrich, St. Louis, MO, USA). The plates were incubated at 37 °C for 24 h and the zone of inhibition were examined. All determinations were undertaken in triplicate and the average zone of inhibition was calculated with the standard deviation.

### 4.11. Determination of the Half-Maximal Inhibitory Concentrations (IC_50_) and the Minimum Inhibitory Concentrations (MICs)

The half-maximal inhibitory concentrations (IC_50_) and the minimum inhibitory concentrations (MICs) of *O. indicum* young fruit extract (OIYF) for each pathogenic bacterium were determined using the broth microdilution method combined with drop plate technique for bacterial enumeration. Briefly, two-fold serial dilutions of extracts in Mueller-Hinton broth (MHB) at concentrations ranging from 0.731–50 mg/mL were placed in a clear, untreated, polystyrene 96-well plate. The bacterial inoculum was prepared from a subculture in blood agar incubated for 18–24 h at 37 °C before to the test. The bacterial culture was adjusted to 0.5 McFarland standard in MHB using a turbidimeter (Biosan, Riga, Latvia). A diluted bacterial suspension was added into the 96-well plate containing the serially diluted extracts to final 1 × 10^6^ CFU/mL. The final volume of 200 μL suspension per well was set. Negative and positive growth controls were performed by adding only MHB and each bacterium with MHB to the wells, respectively. Amoxycillin and doxycycline 4 μg/mL (Sigma-Aldrich, St. Louis, MO, USA) was used as antibiotic control. The plates were incubated at 37 °C for 18–24 h. Then, an optical density (OD) at 600 nm was determined using spectrophotometer (SPECTROstar^®^ Nano, BMG LABTECH, Cary, NC, USA) and the concentration at which no bacterial growth comparing to controls was examined, due to the turbidity interfering with the extracts at a certain concentration range. The capability of different extracts to inhibit bacterial growth was confirmed with quantification by drop plate technique for bacterial enumeration. 

### 4.12. Drop Plate Technique for Bacterial Enumeration (DP)

After the incubation time was complete, all bacterial suspensions were adjusted to approximately desired log10 CFU/mL using standard serial 10-fold dilution in MHB, and 20 µL were transferred for drop plating on Mueller-Hinton agar (*S. intermedius*, *P. aeruginosa*, *β*-hemolytic *E. coli*, and *S. aureus*) or blood agar (*S. Suis*). The drops were absorbed to agar in less than half an hour. Then, the plates were incubated at 37 °C for 18–24 h and the viable pathogens were enumerated. At least 3 to 30 colonies of the bacteria grew from 10 µL of drop and 30 to 300 CFU per 100 µL of the bacteria as a confidence technique were chosen for counting [69]. The total count of CFU from at least three drops at the countable dilution were determined and averaged. Finally, the total count was scaled up and the viable cell counts were expressed as CFU/mL [70]. The bacterial cell viability, the half-maximal inhibitory concentrations (IC_50_) and the minimum inhibitory concentration (MIC) data were analyzed.

### 4.13. Evaluation of Antibacterial Mechanism of Action of O. indicum by Scanning Electron Microscopic Technique 

The Scanning Electron Microcopy (SEM) method was used to observe bacterial cell morphology. Each bacterial culture suspension was adjusted to final concentration of 3 × 10^6^ CFU/mL in Mueller-Hinton broth with the *O. indicum* young fruit extract (OIYF) at a concentration of 5 mg/mL and without extract (control culture) in parallel. The cell suspensions were incubated at 37 °C for 15 h and were harvested for Scanning Electron Microscopy sample preparations. The bacterial cells were washed three times with sterile deionized water at 2000× *g* centrifugation for 10 min. The cell pellet was collected and fixed by 10% paraformaldehyde (Sigma-Aldrich, St. Louis, MO, USA) and 2.5% gluten-aldehyde (Sigma-Aldrich, St. Louis, MO, USA) solution for 30–60 min. The samples were further dehydrated by a serial concentration gradient of 30%, 50%, 70%, 80%, 90%, and 100% ethanol solutions and acetone. The dehydrated cells were transferred to clean glass coverslip, dried and coated by Sputter coater Q150R ES (Quorum, Tokyo, Japan). All samples were kept dried in CO_2_ critical point dryer until they were examined by ultra-high Resolution Scanning Electron Microscope Hitashi SEM SU8020 (Hitachi High-Tech, Tokyo, Japan). Bacterial cell morphological changes were observed.

### 4.14. Evaluation of Correlations between Antioxidant and Antibacterial Activities and Phytochemical Contents 

The correlations (R) between the parameters including total phenolic content, total flavonoid content and flavone contents and the antioxidant and antibacterial activities were evaluated by a regression method using Microsoft Excel (Microsoft Office Professional Plus 2016—Microsoft Corporation, Redmond, DC, USA).

### 4.15. Statistical Analysis

All measurements were performed in triplicate and the results are expressed as mean ± SD. An independent sample *t*-test was used to compare the means and the least significant difference at *p* < 0.05 was calculated. All analyses were performed using Microsoft Excel (Microsoft Office Professional Plus 2016—Microsoft Corporation, Redmond, DC, USA). The bacterial cell viability, the half-maximal inhibitory concentration (IC_50_) and minimum inhibitory concentration (MIC) data were analyzed by dose-response non-linear regression best curve fit for inhibition activity using GraphPad Prism v.9.0.1 (GraphPad Software, CA, USA) and Calcusyn v.1.1 (Biosoft, Cambridge UK) programs. The data are presented as mean ± SD.

## 5. Conclusions

Extracts from the seeds collected from different locations in Thailand, and young fruits and flowers of *O. indicum* showed in vitro DPPH scavenging effects. The seed and young fruit extracts also promoted in vitro antibacterial activities against clinical isolated bacteria including *S. intermedius*, *S. suis*, *P. aeruginosa*, *β*-hemolytic *E. coli* and *S. aureus*. These results corresponded to our previous studies. The orange crystals and yellow precipitates were obtained from the preparation processes of both seed extracts. The orange-red crystal from the seed collected from Lampang province showed both in vitro antioxidant and antibacterial effects on the tested bacteria while the yellow precipitate from the same source showed only antioxidant activity. The formation process of these orange crystals and yellow precipitates is still unclear. Quantitative analysis of phytochemicals in *O. indicum* samples by spectrophotometric and HPLC techniques showed that they contained different amounts of total phenolic, total flavonoid and three major flavones, baicalin, baicalein and chrysin contents. Young fruit extract, which contained low amounts of flavone contents, still promoted antibacterial effects against the tested bacteria. Regression analysis of the results suggest the correlation between total phenolic and total flavonoid contents with antioxidant and antibacterial effects. Baicalin was found to have a high correlation with inhibitory effects against *β*-hemolytic *E. coli* while the three unidentified peaks, which could be flavones, showed high correlations with an inhibitory effect against *S. intermedius*, *S. suis*, *P. aeruginosa* and *S. aureus*. Scanning Electron Microscopy (SEM) suggested that the mechanism of action of *O. indicum* young fruit extract could be via flavonoid-induced bacterial cell lysis and abnormal cell aggregations. Further studies of the phytochemical and physical characteristics and other related biological activities and the mechanisms of action of the fractions and extracts from *O. indicum* are in process.

## Figures and Tables

**Table 1:** Flavonoid Contents in *O. indicum* Extracts as Determined by high-performance liquid chromatography (HPLC).

Sample	DPPH Assay (EC_50_, μg/mL)	TPC (g% GAE)	TFC (g% QE)	Content (g% in the Extract) *			
				Baicalin + B0	Baicalin	Baicalein	Chrysin
OIRL	25.99 ± 3.30 ^a^	8.20 ± 0.31 ^a^	6.01 ± 0.41 ^a^	12.12 ± 0.02 ^a^	6.82 ± 0.04 ^a^	1.12 ± 0.01 ^a^	1.49 ± 0.01 ^a^
OIYL	54.57 ± 5.76 ^b^	6.94 ± 0.34 ^b^	6.56 ± 0.49 ^b^	7.93 ± 0.06 ^b^	1.62 ± 0.04 ^b^	1.06 ± 0.01^b^	2.34 ± 0.03 ^b^
OISL	122.63 ± 4.88 ^c^	5.95 ± 0.76 ^b,c,d^	4.04 ± 0.72 ^c,d^	6.87 ± 0.11 ^c^	6.87 ± 0.11 ^a^	0.98 ± 0.00 ^c^	1.31 ± 0.01 ^c^
OIRP	70.52 ± 0.80 ^d^	1.94 ± 0.00 ^e^	4.78 ± 0.18 ^c^	4.70 ± 0.00 ^d^	2.97 ± 0.03 ^c^	0.32 ± 0.01 ^d^	0.25 ± 0.00 ^d^
OIYP	29.16 ± 0.07 ^a^	7.88 ± 0.17 ^a^	8.74 ± 0.19 ^e^	15.29 ± 0.02 ^e^	1.83 ± 0.04 ^d^	1.06 ± 0.01 ^b^	1.48 ± 0.00 ^a^
OISP	47.87 ± 1.10 ^b^	4.53 ± 0.31 ^c^	3.98 ± 0.21 ^d^	16.24 ± 0.05 ^f^	3.66 ± 0.01 ^e^	2.10 ± 0.01 ^e^	2.24 ± 0.01 ^b^
OIYF	164.41 ± 9.87 ^e^	4.68 ± 0.37 ^c, d^	5.16 ± 0.16 ^c^	0.40 ± 0.00 ^g^	0.19 ± 0.00 ^f^	0.36 ± 0.00 ^d^	1.12 ± 0.00 ^e^
OIFL	180.24 ± 2.20 ^e^	3.78 ± 0.29 ^d^	0.58 ± 0.14 ^f^	0.09 ± 0.00 ^h^	0.04 ± 0.00 ^g^	0.06 ± 0.00 ^f^	0.26 ± 0.00 ^d^
Ascorbic acid	4.04 ± 0.20	-	-	-	-	-	-

* Different letters in the same column are significantly different (*p* < 0.05). DPPH = 2,2-diphenyl-1-picrylhydrazyl, TPC = total phenolic content, TFC = total flavonoid content, B0 = compound B0 which appeared as a peak at the retention time of 6.00 min analyzed by the same HPLC method, OIRL = orange-red crystals from *O. indicum* seed collected from Lampang province, OIYL = yellow precipitates from *O. indicum* seed collected from Lampang province, OISL = *O. indicum* seed extracts from Lampang province, OIRP = orange-red crystals from *O. indicum* seed collected from Pattani province, OIYP = yellow precipitates from *O. indicum* seed collected from Pattani province, OISP = *O. indicum* seed extracts from Pattani province, OIYF = *O. indicum* young fruit extract, OIFL = *O. indicum* flower extract.

**Table 2:** HPLC retention Times, UV Spectra, Previous Reported MS Data and Chemical Structures of Chemical Compounds in *O. indicum* Extracts and Fractions.

Peak No.	Rt (min.)	UV Spectra (λ_max_, nm)	Previous Reported MS Data ^#^ (m/z)	Compound	Chemical Structure
1	4.09	215, 276, 315		compound Rt 4	
		214.9, 277.4, 316.6 ^d^	593, 269 ^b^	oroxin B * (baicalein-7-*O*-diglucoside)	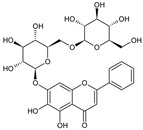
2	6.00	214, 276, 316		compound B0	
		214.5, 277, 316.6 ^d^	431, 269, 251, 241, 223, 195 ^b^	oroxin A * (baicalein7-*O*-glucoside)	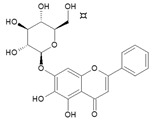
3	6.17	214, 276, 316	269, 445, 891 ^a^	Baicalin (baicalein-7-*O*-glucuronide)	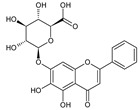
4	7.42	217, 266, 303		compound Rt 7	
		267.9, 305.9 ^d^	429, 253, 209, 175, 113 ^b^	Chrysin-7-*O*-glucuronide *	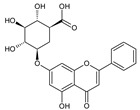
5	9.92	214, 273, 321	223, 251, 269 ^a^	baicalein	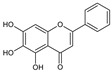
6	12.97	214, 261, 311	180, 181, 209, 253, 254 ^a^	chrysin	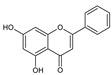

^a^ = Rojsanga et al., 2017 [24], ^b^ = Peng et al., 2019 [13], ^c^ = Lin and Harnly, 2007 [26], ^d^ = Yuan et al., 2008 [18], * proposed compound, ^#^ Mass Spectral Data.

**Table 3:** Antibacterial Effects Against Clinical Isolated Zoonotic Bacteria of *O. indicum* Samples.

Sample	Zone of Inhibition at the Concentration of 50 mg/mL (mm)				
	*S. intermedius*	*S. suis*	*P. aeruginosa*	*β*-Hemolytic *E. coli*	*S. aureus*
OIRL	9.50 ± 0.70	0	0	20.00 ± 0.00	0
OIYL	0	0	0	0	0
OISL	17.00 ± 2.78	11.83 ± 2.25	11.50 ± 0.70	15.75 ± 1.77	10.50 ± 1.32
OIYF	12.83 ± 0.76	10.42 ± 2.13	10.25 ± 1.06	13.00 ± 1.41	11.33 ± 1.26
AMC30	31.00 ± 1.73	25.60 ± 0.96	0	22.00 ± 1.73	25.60 ± 0.96
DO30	15.67 ± 2.31	16.83 ± 1.29	14.25 ± 0.35	25.00 ± 1.41	16.83 ± 1.29
SXT25	0	0	14.50 ± 0.70	25.67 ± 0.58	0

OIRL = orange-red crystals from *O. indicum* seed collected from Lampang province, OIYL = yellow precipitates from *O. indicum* seed collected from Lampang province, OISL = *O. indicum* seed extracts from Lampang province, and OIYF = *O. indicum* young fruit extract. AMC 30 = Amoxicillin/clavulanic acid 30 μg, DO 30 = doxycycline 30 μg and SXT 25 = sulfamethoxazole-trimethoprim 25 μg.

**Table 4:** Minimum Inhibitory Concentrations (MICs) and Half-Maximal Inhibitory Concentration (IC_50_) of *O. indicum* Young Fruit Extract (OIYF) Against Clinical Isolated Zoonotic Bacteria.

Bacterial Species	IC_50_ (mg/mL)	MIC (mg/mL)
*S. intermedius*	0.81 ± 0.04	5.17 ± 1.23
*S. suis*	0.96 ± 0.46	10.54 ± 1.00
*P. aeruginosa*	3.59 ± 1.01	46.72 ± 1.94
*β*-hemolytic *E. coli*	6.57 ± 0.69	19.11 ± 0.62
*S. aureus*	0.40 ± 0.00	4.67 ± 0.04

**Table 5:** The R Values (Correlation Coefficients) between the Antioxidant (1/EC_50_, μg/mL) and Antibacterial Activities (Zone of Inhibition, mm) and Total Phenolic Contents (g% GAE), Total Flavonoid Contents (g% QE) and the Peak Areas of flavones in *O*. *indicum* samples.

	1/DPPH (2,2-Diphenyl-1-Picrylhydrazyl)	*S. intermedius*	*S. suis*	*P. aeruginosa*	*β*-Hemolytic *E. coli*	*S. aureus*
TPC	0.698	0.441	0.838	0.842	0.116	0.890
TFC	0.689	0.925	0.920	0.917	0.505	0.862
B1	0.665	0.545	0.055	0.049	0.774	0.043
B2	0.455	0.208	0.439	0.446	0.022	0.562
C	0.196	0.164	0.433	0.441	0.050	0.557
B1 + B2 + C	0.593	0.023	0.064	0.072	0.066	0.203
B1 + B0	0.573	0.406	0.827	0.830	0.171	0.874
B0	0.556	0.940	0.957	0.956	0.516	0.920
Rt 4	0.574	0.488	0.785	0.790	0.063	0.863
Rt 7	0.598	0.316	0.790	0.791	0.344	0.793

TPC = total phenolic content (g% GAE), TFC = total flavonoid content (g% QE), B1 = baicalin content (g%), B2 = baicalein content (g%), C = chrysin content (g%), B0 = compound at retention time of 6.00 min, Rt 4 = compound at retention time of 4.09 min, Rt 7 = compound at retention time of 7.42 min.

## Data Availability

Data sharing not applicable.

## Sample Availability

Samples of the compounds are not available from the authors.
